# Relationship between epileptiform discharges and social reciprocity or cognitive function in children with and without autism spectrum disorders: An MEG study

**DOI:** 10.1111/pcn.13093

**Published:** 2020-07-19

**Authors:** Tetsu Hirosawa, Paul F. Sowman, Mina Fukai, Masafumi Kameya, Daiki Soma, Shoryoku Hino, Tatsuru Kitamura, Kyung‐min An, Yuko Yoshimura, Chiaki Hasegawa, Daisuke Saito, Takashi Ikeda, Mitsuru Kikuchi

**Affiliations:** ^1^ Department of Psychiatry and Neurobiology, Graduate School of Medical Science Kanazawa University Kanazawa Japan; ^2^ Department of Cognitive Science, Australian Hearing Hub Macquarie University Sydney Australia; ^3^ Department of Neuropsychiatry Ishikawa Prefectural Takamatsu Hospital Kahoku Japan; ^4^ Research Center for Child Mental Development Kanazawa University Kanazawa Japan; ^5^ Faculty of Education, Institute of Human and Social Sciences Kanazawa University Kanazawa Japan

The prevalence of epilepsy in children with autism spectrum disorder (ASD) is reportedly higher than that among typically developing (TD) children.^1^ Many patients with epilepsy meet the diagnostic criteria of ASD.^2^ Even in the absence of clinical seizures, the electroencephalograms of patients with ASD show epileptiform abnormalities (interictal epileptiform discharges [IED]) more often than do those of TD children.^3^ These facts imply an association between ASD and epilepsy. Nevertheless, information related to the possible effects of IED on behavior and cognitive functioning remains scarce. That related to the association between epileptiform discharge and autistic symptomatology is even scarcer.

As one might readily infer, assessing the ongoing effects of IED on autistic social impairment in children with ASD is important to elucidate how IED and autistic symptomatology are related. Furthermore, from a clinical perspective, it would be of interest to ascertain whether the IED at the initial assessment predict later intelligence. Therefore, we specifically examined the association between the frequency of IED and social impairment or intelligence over the course of time in children with and without ASD.

We recruited 40 TD children and 26 children with ASD and excluded participants who had had a clinical diagnosis of any other neuropsychiatric disorder, including epilepsy. We also excluded those who were receiving antiepileptic drugs. At the initial assessment, we recorded magnetoencephalographic (MEG) data for 10 min and evaluated intelligence using the Kaufman Assessment Battery for Children (K‐ABC).^4^ For children in the ASD group, we also measured social impairment using the Social Responsiveness Scale gender‐normed T scores (SRS‐T).^5^ Participants completed a second K‐ABC evaluation (and SRS for the ASD group) after at least 300 days. For each participant, the IED were counted manually by application of the same general principles recommended by the International Federation of Clinical Neurophysiology (Fig. S1). Appendix S1 presents detailed information related to the methodology.^6^

Differences between TD and ASD in terms of age, sex, cognitive performance, frequency (i.e., number of IED per 10 s), prevalence of IED at first measurement, and duration between first and second assessment were assessed (Table S1). Additional statistical analyses were conducted after excluding a female child with ASD who was unable to complete either the K‐ABC or the MEG recording. To predict Mental Processing Scale (MPS) and Achievement Scale (ACH) scores or SRS‐T scores from a linear mixed‐effects analysis, we incorporated the frequency of IED at first measurement, number of days between first and second evaluation, their two‐way interaction, sex, and age at the first assessment into the models as fixed effects. As a random effect, we used intercepts for subjects. Appendix S1 presents more detailed information related to the results.

In children with ASD, we found a significant main effect of the frequency of IED for SRS scores (*z* = −4.3, *P* < 0.001; see Table S2 for more details), and significant interaction effects between the number of days between measurements and the IED frequency for both MPS (estimated fixed effect was 0.01, *z* = 2.57, *P* = 0.10) and ACH (estimated fixed effect was 0.02, *z* = 4.2, *P* < 0.001) scores (Table S3 presents additional details). First, the results implied that higher frequency of IED at the baseline was significantly associated with better social reciprocity. This result resembles those obtained from a study by Hartley‐McAndrew and Weinstock^7^ but contradicts results reported by others.^8^, ^9^ Second, the higher frequency of IED was associated with higher intelligence and achievement at later assessments. To visualize these interaction effects, we computed the respective marginal means from predictions of previously fit models for MPS and ACH. The results are presented in Figure 1. In TD children, the effects were not found to be significant (Table S4 presents additional details).

**Fig. 1:**
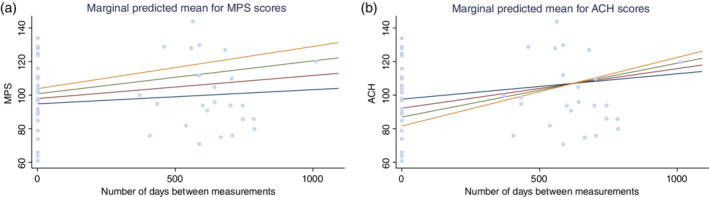
Predictive marginal means of Kaufman Assessment Battery for Children (K‐ABC) scores. The horizontal axes show the number of days between measurements; the vertical axes show (a) Mental Processing Scale (MPS) and (b) Achievement Scale (ACH) scores. The light blue discs represent the observed data points. Each colored line represents predicted marginal mean for the fixed frequency of interictal epileptiform discharges (IED). The lines represent marginal means for fixed IED frequencies. For example, the predicted marginal mean for ‘frequency of IED = 1.0’ (green) represents the estimated mean of the MPS or ACH score where every observation is treated as if it represented individuals who had a frequency of IED of 1.0 and given the number of days between measurements, but represents sex and age at the first measurement as they were observed (see Appendix S1 for details). (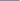) Frequency of IEDs = 0. (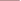) Frequency of IEDs = 0.5. (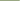) Frequency of IEDs = 1.0. (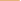) Frequency of IEDs = 1.5. (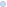) Observed data points.

This report is the first of a study finding that higher frequency of IED predicts better intelligence or achievement at a later assessment. Considering a recent report suggesting the importance of sociality for development of intelligence in children with ASD,^10^ the observed association between IED and intelligence might be driven by the positive effect of IED on sociality. It is noteworthy that if IED in ASD are detrimental, or if they cause cognitive or social dysfunction, then a higher IED frequency can be expected to correspond to lower sociality or intelligence. Regarding results obtained from the current study, that hypothesis is unlikely. Rather, it is apparently protective against primary changes in ASD. However, drawing such a firm conclusion based purely on our results from such a small sample size is inappropriate. Further research with greater statistical power and considering locations and types of IED might be necessary to verify this hypothesis.

Parents agreed to the participation of their children. Written informed consent for participation and publication was obtained before participation. The Ethics Committee of Kanazawa University Hospital approved the methods and procedures, all of which were performed in accordance with the Declaration of Helsinki. The datasets used or analyzed during the current study are available from the corresponding author on reasonable request.

## Disclosure statement

The authors declare that no conflict of interest exists in relation to this paper or the study it describes. They have no biomedical financial interest.

## Supporting information


**Appendix S1.** Supporting information.
**Table S1.** Participant characteristics.
**Table S2.** Effects of IED frequency on change over time in social reciprocity in children with ASD.
**Table S3.** Effects of IED frequency on change over time in intelligence – children with ASD.
**Table S4.** Effects of IED frequency on change over time in intelligence – TD children.

**Figure S1.** An example of observed interictal epileptiform discharges (IED), where IED were defined as sharp transient and clearly different from background activity with an ‘epileptiform’ morphology and a logical spatial distribution (left). Magnetoencephalogram 2‐D topography shows a clear pattern of sink (green) and source (red).
