# Supplementary material for: Relationship between epileptiform discharges and social reciprocity or cognitive function in children with and without autism spectrum disorders: An MEG study
Source: Psychiatry Clin Neurosci. 2020 Jul 19;74(9):510–1. doi: 10.1111/pcn.13093 (PMC7497246; doi:10.1111/pcn.13093)
Supplement: Supplementary file 1 — Appendix S1. Supporting information. Table S1. Participant characteristics. Table S2. Effects of IED frequency on change over time in social reciprocity in children with ASD. Table S3. Effects of IED frequency on change over time in intelligence – children with ASD. Table S4. Effects of IED frequency on change over time in intelligence – TD children. [file PCN-74-510-s001.docx]

**Supporting Information**

**Contents**

**Methods**

**Participants**

**Intelligence assessment**

**Assessment of social reciprocity in children with ASD**

**MEG Recordings**

**Assessment of MEG recordings**

**Statistical analysis**

**Visualization of interaction effects between the frequency of IEDs and number of days between measurements**

**Results**

**Differences of population descriptors between groups**

**Effects of IED frequency on change over time in social reciprocity in children with ASD**

**Effects of IED frequency on change over time in intelligence – children with ASD**

**Effects of IED frequency on change over time in intelligence – TD children**

**Methods**

*Participants*

From Kanazawa University and its affiliated hospitals, we recruited 40 typically developing children (29 male, 11 female; age 32–72 months) and 26 children (21 male, 5 female; age 40–92 months) with ASD. The ASD diagnosis was made according to the Diagnostic and Statistical Manual of Mental Disorders (4th edition) (DSM-IV)^1^ using the Diagnostic Interview for Social and Communication Disorders (DISCO)^2^ or the Autism Diagnostic Observation Schedule – Generic (ADOS-G).^3^ We excluded participants who had clinical diagnosis of any other neuropsychiatric disorder including epilepsy. We also excluded those who were receiving antiepileptic drugs. Parents agreed to the participation of their children. Written informed consent for study participation was obtained from all parents before participation. The Ethics Committee of Kanazawa University Hospital approved the methods and procedures, all of which were performed in accordance with the Declaration of Helsinki. We evaluated cognitive function using the Kaufman Assessment Battery for Children (K-ABC). Participants completed a second evaluation after at least 300 days (range 348–1631 days).

We are continually recruiting participants in a single, large project (Bambi plan, http://bambiplan.w3.kanazawa-u.ac.jp/pdf/jusen_english.pdf). Some participants and MEG recordings overlap with our earlier studies.^4–6^ However, the results of the second evaluation do not. Furthermore, the emphases of those earlier studies differed from those of this study.

*Intelligence assessment*

Actually, K-ABC was developed to distinguish problem-solving abilities and knowledge of facts. The former set of skills is interpreted as intelligence. The latter is defined as achievement.^7^ In K-ABC, the problem solving abilities are measured using the Mental Processing Scale (MPS). The achievement is measured according to the Achievement scale (ACH). These scores, which are provided as age-adjusted standardized scores, are normalized to have a mean of 100 and a standard deviation of 15.^8^ Haddad and Naglieri suggested that K-ABC differed from the Wechsler Intelligence Scale for Children – Revised (WISC-R;^9^): it measures intelligence and achievement separately; language plays a minimal role in the measurement of intelligence; and the child has sufficient time to learn how to solve the presented tasks.^10^ Reported correlations of MPS with Full Scale IQ measured using WISC-R tend to be moderate (0.62–0.76).^11^ Therefore, the intelligence measured using the K-ABC resembles the intelligence measured by the WISC-R, but their measured phenomena are not identical. They have their own unique characteristics.

*Assessment of social reciprocity in children with ASD*

In the ASD group, the parent(s) of the participants filled out the Social Responsiveness Scale (SRS). We used gender-normed total T scores (SRS-T).^12^ Higher scores represent lower social reciprocity. Although the validity of self-ratings of children is still under assessment, the SRS can be completed by a parent, a teacher, or another adult informant. In this way, it involves ratings of children in their natural social contexts and reflects what has been observed consistently over weeks or months of time rather than merely reflecting results of a single clinical or laboratory observation.^13^ Therefore, it capitalizes on both direct observation and on the accumulated history of behaviors observed by the informant over time. By virtue of this characteristic, SRS reflects social abilities that are apparent not only in one-to-one but also in one-to-many or in many-to-many real-world communication, rather than those appearing in one-to-one communications or in virtual communications in an examination room. As such, for comparison with other parents or teacher-reported ASD-directed behavior assessments, good agreement has been reported (e.g., SCQ^14–16^ Children’s Communication Checklist^15–17^), and Social and Communication Disorders Checklist.^18^ The SRS scores are also known to exhibit high inter-rater reliability ^19^ and are known to be distributed continuously in a general population.^20^

*MEG Recordings*

The MEG recording methods used for this study were identical to those used for our earlier study.^25^ We recorded MEG data using a 151-channel Superconducting Quantum Interference Device, whole-head coaxial gradiometer MEG system for children (PQ 1151R; Yokogawa/KIT, Kanazawa, Japan) in a magnetically shielded room (Daido Steel Co., Ltd., Nagoya, Japan) installed at the MEG Center of Ricoh Co. Ltd. (Kanazawa, Japan). The custom child-sized MEG system facilitates measurement of brain responses in young children, which would otherwise be difficult using conventional adult-sized MEG systems. The child-sized MEG system ensures that the sensors are positioned easily and effectively for the child's brain. Moreover, it ensures that head movements are constrained.^21^

The band-pass-filtered MEG data (0.16–200 Hz) were collected at a sampling rate of 1,000 Hz. During MEG recording, one staff member escorted each participant into the shielded room, which had been decorated with colorful pictures of Japanese (cartoon) characters and colorful pictures resembling an attractive vehicle adopted from an animation series that is popular with preschool children. During measurements, the staff member stayed in the shielded room, comforting and encouraging each participant to maintain a steady body position when necessary. Parent(s)/caretaker(s) were able to observe the child via a TV monitor during measurements. During MEG recording, the children lay supine on a bed and viewed a video program projected onto a screen (i.e., eyes-open condition). The head position within the helmet during the MEG recording was adjusted while measuring the magnetic fields after passing currents through coils attached at three locations on the head surface, which served as fiduciary marks for the bilateral mastoid processes and nasion. Before recording, we prepared several video programs that were entertaining for young children. Each participant was shown a video program that they had selected. Before recording, each child confirmed that the video program contents had been selected. Then MEG was recorded for 600 s. The MEG recording was done between 11 a.m. and 3 p.m. No child showed a clear sign of drowsiness in terms of MEG waveforms.

*Assessment of MEG recordings*

The assessment procedures for MEG recordings were identical to those used in our earlier study.^22^ One investigator (author T.H.) reviewed the raw MEG signals (i.e., time vs. amplitude waveforms). He had been trained in EEG/MEG and epilepsy for 11 years and had extensive experience in distinguishing interictal epileptiform discharges (IEDs) from other non-epileptic waveforms. The IEDs were detected manually by application of the same general principles recommended by the International Federation of Clinical Neurophysiology^23^ and were used in standard EEG interpretation: The sharp transient is clearly different from background activity with an “epileptiform” morphology and a logical spatial distribution (e.g., Figure S1). We did not include the locations of the foci in the statistical analysis because of the small sample size. After counting of IEDs during the 600 s MEG recording for each participant, we calculated the frequency of IEDs (i.e., number of IEDs per 10 s) for each.

*Statistical analysis*

Differences in population descriptors between TD and ASD were tested using Student *t*-tests for age and cognitive performance (i.e., MPS and ACH in K-ABC), and using Mann–Whitney U-tests for the frequency of IEDs and duration between first and second assessment. Chi-square tests were used for analyses of the prevalence of IEDs and sex.

We specifically examined the frequency of IEDs rather than its prevalence because frequency of IEDs is more important than its mere existence if one considers that IEDs might affect cognitive dysfunction. To explore the relation between the frequency of IEDs and social or cognitive functioning as well as its effects on later cognitive and social functioning, we conducted a linear mixed effects analysis. Before assessing the results from that analysis, we verified how well our data met the assumptions for the models. Specifically, we verified the following assumptions for each analysis: linearity, normality, homogeneity of variance, model specification, influence, collinearity, and Gaussian distribution of overall error distribution in each model. Among those, only homogeneity of variance was violated (we checked the graphical representations and the Breusch–Pagan test). We chose to use heteroscedasticity-robust standard errors.^24^

To predict MPS and ACH scores or SRS-T scores, because of a possible influence of sex and age^25–27^, we added those in the model to control for the confounding effects. Consequently, against the ACH or MPS scores in K-ABC, or SRS-T, we incorporated the IED frequency, number of days between first and second evaluation, their two-way interaction and sex (without an interaction term) and age at the first assessment (without an interaction term) into the models as fixed effects. As a random effect, we used intercepts for subjects. We applied this model respectively to data of children with ASD and to data of TD children.

For analyses of the scores of K-ABC and SRS in children with ASD, statistical significance was inferred for *p*< .0167 after Bonferroni correction for three significance tests (i.e., MPS, ACH, and SRS). For analyses of the scores of K-ABC in TD children, statistical significance was inferred for *p*< .025 after Bonferroni correction for two significance tests. All statistical analyses were conducted using software (Stata ver. 15.0; Stata Corp., College Station, TX, USA).

*Visualization of interaction effects between the frequency of IEDs and number of days between measurements*

To visualize those significant interaction effects, we computed the marginal means from predictions of the previously fit linear mixed models, respectively, for MPS and ACH. Specifically, we set values for frequency of IEDs as 0, 0.5, 1.0, and 1.5 (per 10 min) and set the number of days between measurements as 0, 182.5, 365, 547.5, 730, 912.5, and 1095. Then, for each pair of values, we predicted the marginal mean of MPS or ACH based on the previously fitted models. Therefore, in predicting MPS or ACH, we fixed the frequency of IEDs and number of days between measurements, but the remaining variables (i.e., sex and age at the first assessment) were allowed to vary as they were observed. For example, the predicted marginal mean for “frequency of IEDs = 0.5, number of days between measurement = 365” is the estimated mean of the dependent variable (i.e., MPS or ACH), where every observation is treated as if it represents individuals who have a frequency of IEDs of 0.5 and number of days between measurement of 365, but represent sex and age at the first measurement as they are observed.

**Results**

*Differences of population descriptors between groups*

One female TD child and one child with ASD were unable to complete the MEG recording. The child with ASD was also unable to complete K-ABC because of severe psychomotor agitation. Two of the female TD children were not older than the minimum age requirement for ACH. A significant difference was found between TD and ASD groups in terms of age at the first assessment and the duration between the first and second assessment. Table S1 presents these results. Although both the prevalence (18% vs. 39%) and the frequency of IEDs (0.03 vs. 0.13 per 10 s) were higher among children with ASD, neither was statistically significant. Considering earlier reports describing significant difference^22,28–30^, the statistical power derived from this sample size (i.e., 40 vs. 26) might be insufficient to infer the difference as significant.

Further statistical analyses were conducted after excluding the female child with ASD who was unable to complete both the K-ABC and the MEG recording.

*Effects of IED frequency on change over time in social reciprocity in children with ASD*

A significant main effect was found for the IED frequency (*r* = -11.9, *z* = -4.3, *p*< .05), implying a negative association between the IED frequency and the scores in SRS-T. Higher frequency of IEDs was significantly associated with better social reciprocity. It is noteworthy that higher scores in SRS-T represent lower social reciprocity. No other factor was found to be significant (Table S2).

*Effects of IED frequency on change over time in intelligence – children with ASD*

For the ASD group, a significant interaction effect was found between the number of days between measurement and the IED frequency at first measurement for both MPS (*z* = 2.50 *p*< .0167) and ACH (*z* = 4.2 *p*< .0167) scores. No other factor was found to be significant (Table S3). Those results implied that higher frequency of IEDs at the baseline was associated with higher MPS or ACH at later assessments compared with that in the initial assessment. To visualize these interaction effects, we computed marginal means from predictions of previously fit models for MPS and ACH, respectively.

*Effects of IED frequency on change over time in intelligence – TD children*

For the TD group, we found a significant effect of sex for the MPS score. No other factor was found to be significant (Table 3). A sex difference in MPS and ACH in TD children is consistent with existing data showing more rapid mental development for girls than for boys at the preschool age level.^26,27^ This sex difference is also known to be non-existent among school age children.^27^

**List of abbreviations**

ACH, Achievement Scale; ADOS-G, Autism Diagnostic Observation Schedule – Generic; ASD, autism spectrum disorder; DISCO, Diagnostic Interview for Social and Communication Disorders; DSM-IV, Diagnostic and Statistical Manual of Mental Disorders, fourth edition; EEG, electroencephalography; IEDs, interictal epileptiform discharges; K-ABC, Kaufman Assessment Battery for Children; MEG, magnetoencephalography; MPS, Mental Processing Scale; SRS-T, Social Responsiveness Scale, gender-normed T score; TD, typically developing

***References***

1. American Psychiatric Association [APA]. Diagnostic and Statistical Manual of Mental Disorders, Fourth Edition, Text Revision (DSM-IV-TR). American Psychiatric Association. 2000.

2. Wing L, Leekam SR, Libby SJ, Gould J, Larcombe M. The Diagnostic Interview for Social and Communication Disorders: Background, inter-rater reliability and clinical use. *J. Child Psychol. Psychiatry Allied Discip.* 2002; **43**: 307-125.

3. Lord C, Risi S, Lambrecht L, Cook EH, Leventhal BL, Dilavore PC et al. The Autism Diagnostic Observation Schedule – Generic: A standard measure of social and communication deficits associated with the spectrum of autism. *J. Autism Dev. Disord*. 2000; **30**: 205-123.

4. Remijn GB, Kikuchi M, Shitamichi K, Ueno S, Yoshimura Y, Nagao K et al. Somatosensory Evoked Field in Response to Visuotactile Stimulation in 3- to 4-Year-Old Children. *Front Hum. Neurosci.* 2014; **8**: 170.

5. Yoshimura Y, Kikuchi M, Ueno S, Shitamichi K, Remijn GB, Hiraishi H et al. A longitudinal study of auditory evoked field and language development in young children. *Neuroimage*. 2014; **101**: 440-447.

6. Yoshimura Y, Kikuchi M, Shitamichi K, Ueno S, Remijn GB, Haruta Y et al. Language performance and auditory evoked fields in 2- to 5-year-old children. *Eur. J. Neurosci.* 2012; **35**: 644-650.

7. Kaufman, AS, Kaufman N. Kaufman Assessment Battery for Children. Circle Pines, MN: American Guidance Service; 1983.

8. Kaufman assessment battery for children. *J. Pediatr. Psychol.* 1985; **10**: 257-259.

9. Wechsler D. Wechsler Intelligence Scale for Children-Revised (WISC-R), New York: Psychological Corporation. 1974.

10. Kaufman AS, O’neal MR, Avant AH, Long SW. Review Article: Introduction to the Kaufman Assessment Battery for Children (K-ABC) for Pediatric Neuroclinicians. *J. Child Neurol*. 1987; **2**: 3-16.

11. Kaufman AN. Kaufman Assessment Battery for Children: Administration and Scoring Manual: American Guidance Service. Minneapolis, MN; 1983.

12. Constantino JN, Davis SA, Todd RD, Schindler MK, Gross MM, Brophy SL et al. Validation of a brief quantitative measure of autistic traits: Comparison of the social responsiveness scale with the Autism Diagnostic Interview – Revised. *J. Autism Dev. Disord*. 2003; **33**: 427-433.

13. Volkmar F. Encyclopedia of Autism Spectrum Disorders. Springer, New York, NY, 2013.

14. Rutter M, Bailey A, Lord C. The social communication questionnaire: Manual. Western Psychological Services, Torrance, CA; 2003.

15. Charman T, Baird G, Simonoff E, Loucas T, Chandler S, Meldrum D et al. Efficacy of three screening instruments in the identification of autistic-spectrum disorders. *Br. J. Psychiatry*. 2007; **6**: 554-559.

16. Pine DS, Guyer AE, Goldwin M, Towbin KA, Leibenluft E. Autism spectrum disorder scale scores in pediatric mood and anxiety disorders*. J. Am. Acad. Child Adolesc. Psychiatry*. 2008; **47**: 652-661.

17. Bishop DVM. Development of the Children’s Communication Checklist (CCC): A method for assessing qualitative aspects of communicative impairment in children*. J. Child Psychol. Psychiatry Allied Discip*. 1998; **39**: 879-891.

18. Bölte S, Westerwald E, Holtmann M, Freitag C, Poustka F. Autistic traits and autism spectrum disorders: The clinical validity of two measures presuming a continuum of social communication skills. *J. Autism Dev. Disord*. 2011; **41**: 66-72.

19. Constantino JN, Yang D, Gray TL, Gross MM, Abbacchi AM, Smith SC et al. Clarifying the associations between language and social development in autism: A study of non-native phoneme recognition. *J. Autism Dev. Disord*. 2007; **37**: 1256-1263.

20. Constantino JN, Todd RD. Autistic Traits in the General Population. *Arch. Gen. Psychiatry*. 2003; **60**: 524-530.

21. Tesan G, Johnson BW, Reid M, Thornton R, Crain S. Measurement of neuromagnetic brain function in pre-school children with custom sized MEG. *J. Vis. Exp.* 2010; **19**: 36.

22. Hirosawa T, Kikuchi M, Fukai M, Hino S, Kitamura T, An K-M et al. Association Between Magnetoencephalographic Interictal Epileptiform Discharge and Cognitive Function in Young Children With Typical Development and With Autism Spectrum Disorders. *Front Psychiatry*. 2018; **19**: 568.

23. WA C. IFCN Recommendations for the practice of clinical neurophysiology Amsterdam. Elsevier, editor. Amsterdam; 1983.

24. White H. A Heteroskedasticity-Consistent Covariance Matrix Estimator and a Direct Test for Heteroskedasticity. *Econometrica*. 1980; **48**: 817-838.

25. Baumbach HD, Kao Liang Chow. Visuocortical epileptiform discharges in rabbits: Differential effects on neuronal development in the lateral geniculate nucleus and superior colliculus. *Brain Res*. 1981; **209**: 61-76.

26. Ames LI of CD. The Gesell Institute’s Child From One to Six: Evaluating the Behavior of the Preschool Child. Harper & Row, editor. New York; 1979.

27. Burns CW, Reynolds CR. Patterns of sex differences in children’s information processing with and without independence from g. *J. Sch. Psychol*. 1988; **26**: 233-242.

28. Borusiak P, Zilbauer M, Jenke AC. Prevalence of epileptiform discharges in healthy children–new data from a prospective study using digital EEG. *Epilepsia.* 2010; **51**: 1185-1188.

29. Bryson SE, Clark BS, Smith IM. First report of a Canadian epidemiological study of autistic syndromes. *J. Child Psychol. Psychiatry.* 1988; **29**: 433-445.

30. Hughes JR, Melyn M. EEG and seizures in autistic children and adolescents: further findings with therapeutic implications. *Clin. EEG Neurosci*. 2005; **36**: 15-20.

Figure S1

An example of observed interictal epileptiform discharges (IEDs), where IEDs were defined as sharp transient and clearly different from background activity with an “epileptiform” morphology and a logical spatial distribution (left). Magnetoencephalogram two-dimensional topography shows a clear pattern of sink (green) and source (red).

Table S1. Participant characteristics

|  | TD | ASD | *p* |
| --- | --- | --- | --- |
| *n* | 40 | 25 |  |
| Prevalence of IEDs (Negative/Positive) ^†^ | 33 / 6 | 18 / 7 | .22 |
| Frequency of IEDs (per 10 s) ^§^ | 0.03 (0.1) | 0.13 (0.4) | .20 |
| Gender (Male/Female) ^†^ | 29 / 11 | 21 / 4 | .28 |
| Age in Months^‡^ | 48.6 (8.8) | 62.2 (12.9) | <.05* |
| Duration between first and second assessment (day) ^§^ | 824 (41) | 622 (19) | <.05* |
|  |  |  |  |
| K-ABC scores |  |  |  |
| Mental processing scale^‡^ | 100.7 (9.1) | 97.5 (21.6) | .41 |
| Achievement scale^‡^ | 101.1 (15.6) | 97.6 (21.6) | .44 |

Numbers are mean (standard deviation) or counts. One female TD child and one child with ASD were unable to complete the MEG recording. The child with ASD was also unable to complete K-ABC because of severe psychomotor agitation. Two of the female TD children did not meet the minimum age requirement for ACH.

^†^ Chi-square test

^‡^ Student *t*-tests

^§^ Mann–Whitney U-test

* Statistically significant

ASD, autism spectrum disorder; IEDs, interictal epileptiform discharges; K-ABC, Kaufman Assessment Battery for Children; TD, typically developed controls

Table S2

Effects of IED frequency on change over time in social reciprocity in children with ASD

| **SRS-T** |  |  |  |  |  |
| --- | --- | --- | --- | --- | --- |
|  |  |  |  |  |  |
| **variable** | **Coeff..** | **SE** | **z** | **95% CI** | ***p*** |
| Number of days between measurement | -0.01 | 0.009 | -1.1 | -0.28 – 0.01 | .28 |
| Frequency of IEDs (/10 s) | -11.9 | 2.8 | -4.3 | -17.4 – -6.5 | <.001* |
| Number of days between measurement × Frequency of IEDs | 0.006 | 0.009 | 0.6 | -0.01 – 0.02 | .54 |
|  |  |  |  |  |  |
| Sex ( Male =1 / Female = 0 ) | -6.3 | 6.8 | -0.9 | -19.6 – 7.0 | .35 |
| Age (months) | 0.2 | 0.2 | 1.1 | -0.17 – 0.61 | .28 |

* Statistically significant

ASD, autism spectrum disorder; Coeff., regression coefficient; CI, confidence interval; IEDs, interictal epileptiform discharges; SE, robust standard error; SRS-T, social responsiveness scale T score; TD, typically developed controls

Table S3

Effects of IED frequency on change over time in intelligence – children with ASD

| **MPS** |  |  |  |  |  |
| --- | --- | --- | --- | --- | --- |
|  |  |  |  |  |  |
| **variable** | **Coeff.** | **SE** | **z** | **95% CI** | ***p*** |
| Number of days between measurement | 0.008 | 0.01 | 0.8 | 0.01 – 0.03 | .44 |
| Frequency of IEDs (/10 s) | 6.0 | 9.9 | 0.6 | -13.3 – 25.3 | .54 |
| Number of days between measurement × Frequency of IEDs | 0.01 | 0.004 | 2.6 | 0.003 – 0.019 | .010* |
|  |  |  |  |  |  |
| Sex (Male=1 / Female=0) | 12.2 | 10.2 | 1.2 | -7.9 – 32.3 | .23 |
| Age (months) | -0.2 | 0.3 | -0.6 | -0.8 – 0.4 | .52 |
|  |  |  |  |  |  |
| **ACH** |  |  |  |  |  |
|  |  |  |  |  |  |
| **variable** | **Coeff.** | **SE** | **z** | **95% CI** | ***p*** |
| Number of days between measurement | 0.02 | 0.01 | 1.1 | -0.11 – 0.04 | .27 |
| Frequency of IEDs ( /10 s) | -10.6 | 9.7 | -1.1 | -29.5 – 8.34 | .27 |
| Number of days between measurement × Frequency of IEDs | 0.02 | 0.04 | 4.2 | 0.009 – 0.025 | <.001* |
|  |  |  |  |  |  |
| Sex ( Male=1 / Female=0 ) | 2.7 | 13.4 | 0.2 | -23.6 – 28.9 | .84 |
| Age (months) | -0.1 | 0.4 | -0.3 | -0.88 – 0.65 | .76 |

*Statistically significant

ASD, autism spectrum disorder; ACH, achievement scale; Coeff.., regression coefficient; CI, confidence interval; IEDs, interictal epileptiform discharges; MPS, mental processing scale; SE, robust standard error; SRS-T, social responsiveness scale T score; TD, typically developed controls

Table S4

Effects of IED frequency on change over time in intelligence – TD children

| **vs. MPS** |  |  |  |  |  |
| --- | --- | --- | --- | --- | --- |
|  |  |  |  |  |  |
| **variable** | **Coeff.** | **SE** | **z** | **95% CI** | ***p*** |
| Number of days between measurement | -0.003 | 0.005 | -0.63 | -0.014 – 0.007 | .53 |
| Frequency of IEDs (/10 s) | 10.1 | 6.1 | 1.7 | -1.8 – 22.0 | .10 |
| Number of days between measurement × Frequency of IEDs | 0.002 | 0.005 | 0.3 | -0.008 – 0.010 | .75 |
|  |  |  |  |  |  |
| Sex ( Male=1 / Female=0 ) | -9.6 | 3.0 | -3.2 | -15.5 – -3.8 | .001* |
| Age (months) | 0.1 | 0.1 | 0.7 | -0.19 – 0.38 | .50 |
|  |  |  |  |  |  |
| **vs. ACH** |  |  |  |  |  |
|  |  |  |  |  |  |
| **variable** | **Coeff.** | **SE** | **z** | **95% CI** | ***p*** |
| Number of days between measurement | 0.01 | 0.01 | 1.6 | -0.003 – 0.026 | .12 |
| Frequency of IEDs (/10 s) | 1.3 | 6.4 | 0.2 | -11.2 – 13.8 | .84 |
| Number of days between measurement × Frequency of IEDs | -0.008 | 0.005 | -1.6 | -0.02 – 0.02 | .12 |
|  |  |  |  |  |  |
| Sex ( Male=1 / Female=0 ) | -8.8 | 4.1 | -2.1 | -16.8 – -0.73 | .03 |
| Age (months) | -0.4 | 0.2 | -2.1 | -0.80 – -0.01 | .04 |

* Statistically significant

ASD, autism spectrum disorder; ACH, achievement scale; Coeff.., regression coefficient; CI, confidence interval; IEDs, interictal epileptiform discharges; MPS, mental processing scale; SE, robust standard error; SRS-T, social responsiveness scale T score; TD, typically developed controls
