# Supplementary figures and images for: Relationship between epileptiform discharges and social reciprocity or cognitive function in children with and without autism spectrum disorders: An MEG study
Source: Psychiatry Clin Neurosci. 2020 Jul 19;74(9):510–1. doi: 10.1111/pcn.13093 (PMC7497246; doi:10.1111/pcn.13093)

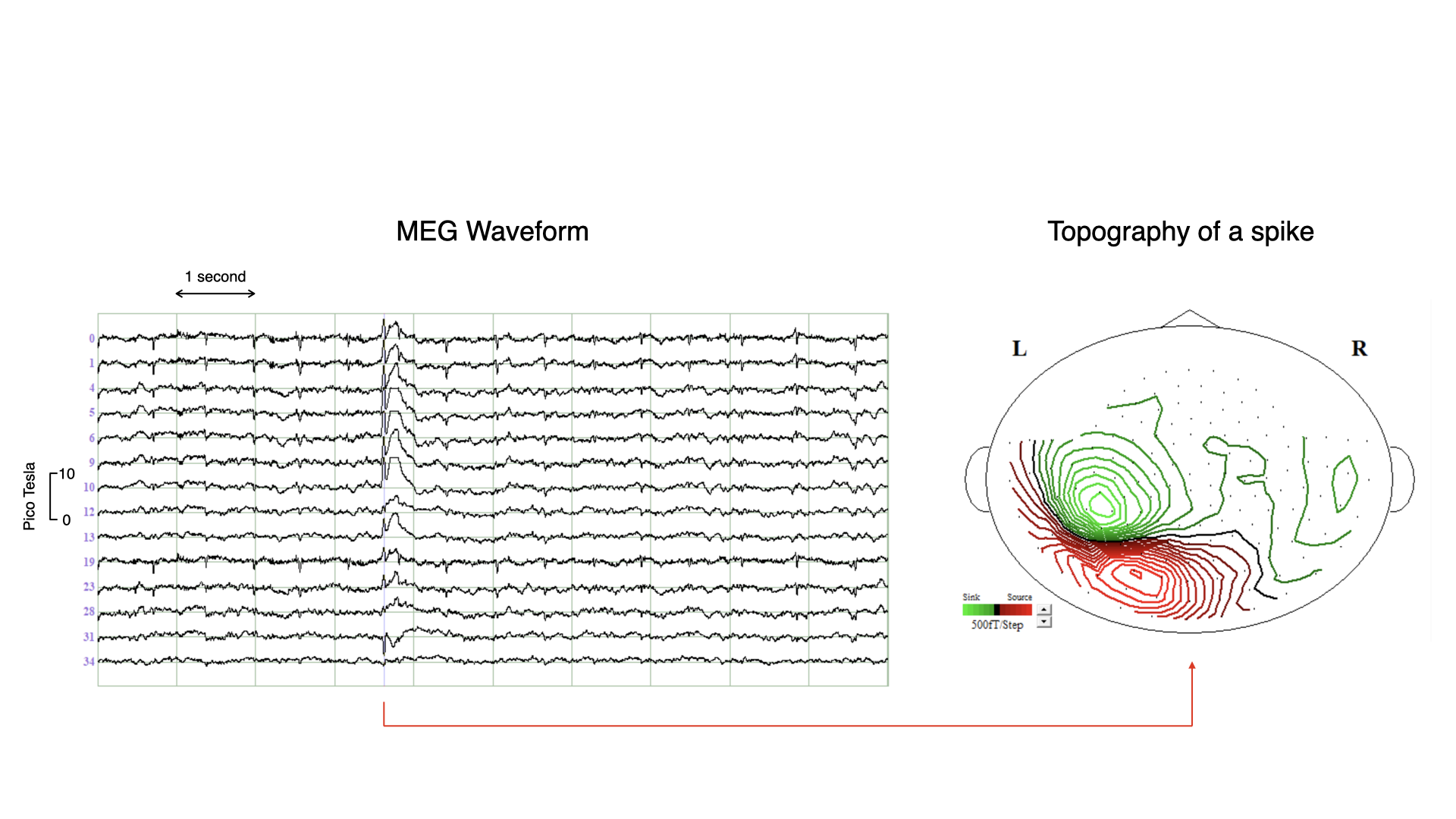

Supplement: Supplementary file 2 — Figure S1. An example of observed interictal epileptiform discharges (IED), where IED were defined as sharp transient and clearly different from background activity with an ‘epileptiform’ morphology and a logical spatial distribution (left). Magnetoencephalogram 2‐D topography shows a clear pattern of sink (green) and source (red). [file PCN-74-510-s002.tif]
